# Rheological and Thermal Conductivity Study of Two-Dimensional Molybdenum Disulfide-Based Ethylene Glycol Nanofluids for Heat Transfer Applications

**DOI:** 10.3390/nano12061021

**Published:** 2022-03-21

**Authors:** Syed Nadeem Abbas Shah, Syed Shahabuddin, Mohammad Khalid, Mohd Faizul Mohd Sabri, Mohd Faiz Mohd Salleh, Norazilawati Muhamad Sarih, Saidur Rahman

**Affiliations:** 1 Department of Mechanical Engineering (Main Campus Lahore), University of Engineering, and Technology, Lahore 54890, Pakistan; 2 Department of Science, School of Technology, Pandit Deendayal Energy University, Knowledge Corridor, Raisan Village, Gandhinagar 382007, Gujarat, India; 3 Graphene & Advanced 2D Materials Research Group, School of Engineering and Technology, Sunway University, No. 5 Jalan University, Bandar Sunway, Subang Jaya 47500, Malaysia; khalids@sunway.edu.my; 4 Department of Mechanical Engineering, Faculty of Engineering, Universiti Malaya, Kuala Lumpur 50603, Malaysia; 5 Department of Electrical Engineering, Faculty of Engineering, Universiti Malaya, Kuala Lumpur 50603, Malaysia; faizsalleh@um.edu.my; 6 Department of Chemistry, Faculty of Science, Universiti Malaya, Kuala Lumpur 50603, Malaysia; 7 Research Center for Nano-Materials and Energy Technology (RCNMET), School of Engineering and Technology, Sunway University, Bandar Sunway, Petaling Jaya 47500, Malaysia; saidur@sunway.edu.my

**Keywords:** nanofluids, molybdenum disulfide, rheology, thermal conductivity, heat transfer

## Abstract

The rheological behavior of two-dimensional (2D) MoS_2_-based ethylene glycol (EG) nanofluids (NFs) was investigated at low volume concentrations (0.005%, 0.0075%, and 0.01%) in a wide temperature range of 0–70 °C and at atmospheric pressure. A conventional two-step method was followed to prepare NFs at desired volume concentrations. Based on the control rotational (0.1–1000 s^−1^ shear rate) and oscillation (0.01–1000% strain) methods, the viscoelastic flow curves and thixotropic (3ITT (three interval thixotropic) and hysteresis loop) characteristics of NFs were examined. Shear flow behavior revealed a remarkable reduction (1.3~14.7%) in apparent dynamic viscosity, which showed concentration and temperature dependency. Such remarkable viscosity results were assigned to the change in activation energy of the ethylene glycol with the addition of MoS_2_. However, the nanofluids exhibited Newtonian behavior at all temperatures for concentrations below 0.01% between 10 and 1000 s^−1^. On the other hand, strain sweep (@1Hz) indicated the viscoelastic nature of NFs with yielding, which varied with concentration and temperature. Besides, 3ITT and hysteresis loop analysis was evident of non-thixotropic behavior of NFs. Among all tested concentrations, 0.005% outperformed at almost all targeted temperatures. At the same time, ~11% improvement in thermal conductivity can be considered advantageous on top of the improved rheological properties. In addition, viscosity enhancement and reduction mechanisms were also discussed.

## 1. Introduction

The soaring demand for energy and fossils fuel emissions requires efficient commercial thermal systems. Profoundly, nanotechnology developments showed great potential to address such issues. The heat exchangers are integral parts of many process and energy production systems. For the last few decades, the suspending particles (size < 100 nm) inside conventional heat transfer fluids demonstrated rational improvement in heat transfer rates [1]. However, nanoparticle suspensions (called nanofluids) were shown to influence the flow behavior when in operation. As reported earlier, apart from the geometrical aspects of heat transfer devices, the density, Reynolds number, and viscosity of the working medium (heat transfer fluid) also altered the pressure drop in the flow loop. With the addition of alumina nanoparticles (1–4 vol%), an increase in pressure drop (5–45%) was witnessed practically. Consequently, there was a corresponding increase in pumping that appeared in a range of 14–90% at Reynold’s number of 1800 [2]. This occurred because of the fluid resistance, which converts a part of mechanical input into heat; thus the internal energy of the fluid system changes [3]. Hence, the true flow properties of nanofluids can provide good guidelines for computations of heat and mass transport mechanisms [4].

Recent research demonstrated that apart from the improvement in thermal performance of nanofluid-based systems, the hydraulic efficiency was affected due to an increase in viscosity, leading to a severe pressure drop [5,6,7,8,9,10]. Kumar and Sarkar reported a maximum increment of ~51.2% in pressure drop due to a rise in viscosity (max ~8.8%) as a result of particle loadings in nanofluids [11]. Another report showed a maximum ~40% increase in friction factor, which caused greater pressure drop for water-based multi-walled carbon nanotube (MWCNT) nanofluids [6]. In contrast, Hussein et al. presented a maximum ~9.9% rise in pressure drop using MWCNT/water nanofluids which varied substantially with concentration (0.075–0.25 mass%) [12]. Sarafraz et al. showed a ~37.5% rise in pressure drop due to friction and viscosity enhancement (with concentration) for iron oxide-based therminol nanofluids [13]. Thermodynamically, flow resistance due to viscosity enhancement causes viscous dissipation, which increases the entropy generation of a thermal system. Consequently, it significantly affects the performance of the thermodynamic system [14].

Moreover, Gaweł Zyła presented the shear thinning (non-Newtonian) and thixotropic behavior of ethylene glycol (EG)-based nanofluids in the presence of carbon black nanoparticles. It was shown that only low mass concentration (0.001%) exhibited the Newtonian profile, whereas viscosity increased monotonically with concentration [15]. Yu et al. reported the transformation from Newtonian to non-Newtonian and thixotropic behavior beyond 0.0571 vol% of MWCNT/water nanofluids [16]. Thus, the adapted viscosity of nanofluids with concentration and temperature in the heat transfer systems and their flow behavior (Newtonian/non-Newtonian/Thixotropic) are of paramount importance while selecting the size and concurrently the design of a pump [17]. Such preliminary knowledge about the flow behavior of nanofluids could add economic benefits by selecting the appropriate size of the pump. This is mainly dependent on the viscous transport behavior of the nanofluids in any engineering system, including heat transport [18]. Therefore, to ameliorate the performance of thermal systems, thermal conductivity enhancement should be inflated in comparison to viscosity [19,20]. In this context, it is vital to perform the rigorous rheological evaluation of any nanofluid over a range of operating parameters (shear rate, temperature, time, etc.) [21].

More recently, graphene analogous 2D nanomaterials such as MoS_2_ are receiving much attention due to their remarkable optoelectronic, lubrication, and thermal properties [22,23,24]. Nikkam et al. reported that the inclusion of MoS_2_ within ethylene glycol can improve thermal conductivity. Their results showed a ~16.4% thermal conductivity improvement at the expense of 9.7% viscosity enhancement at an operating temperature of 20 °C with 1 wt% particle loadings [25]. However, the work remained limited only to 20 °C and tested particle loadings fall in a range of 0.25–1 wt%, which is quite high. The MoS_2_ nanoparticles are quite dense as compared to ethylene glycol so a lower concentration should be preferred to avoid possible sedimentation. Since the thermal systems operate in the wide temperature range, thermo-physical evaluation must be carried out in a temperature range, particularly for flow behavior in a range of shear rates over a wide temperature span.

In view of the foregoing literature, the addition of nanoparticles severely increases the inevitable pressure drop and viscosity which varies with concentration. Consequently, more input pumping power is needed to maintain the flow to achieve the desired heat/energy transfer rates. However, the increased viscosity of nanofluids produced additional viscous effects, which slowed down the fluid movement. Such reduced local velocities lower the temperature gradient and decrease the mass transport, which is the major reason behind the reduced heat/energy transport in flow thermal systems [26]. Therefore, the heat transfer fluids should have higher thermal conductivity and low viscosity to keep balance in the performance of thermal systems [27]. The literature has not focused on the protocols required to mitigate the hydraulic resistance that occurs as a result of particle loadings. Therefore, in the present work, 2D MoS_2_ with lubrication properties was employed to produce a new form of ethylene glycol-based nanofluids with low concentration and improved hydraulic performance. A comprehensive experimental rheological analysis was carried out to investigate the lubrication effect and probe the best concentration of the studied nanofluids over a wide temperature range. Interestingly, the present results unveil the exotic potential of MoS_2_/EG nanofluids in reducing the apparent dynamic viscosity. Thus the anti-friction nanomaterials could be of great interest in heat transfer flow systems to circumvent the enhanced flow resistance of the corresponding nanofluids. The current work emphasizes the rheological behavior of nanofluids. However, thermal conductivity analysis was also carried out.

## 2. Materials and Methods

### 2.1. Materials

The 2D MoS_2_ nanoparticles (CAS-Reg.No.:1317-33-5 MoS_2,_ Blue-Silver Grey Crystalline Solid powder) with an average particle size of 90 nm, density 5.06 (g/cm^3^), and molecular weight 160.08 (g/mol) were procured from lower friction (M K Impex Corp. Mississauga, ON, Canada). Ethylene glycol (EG) with the chemical formula (CH_2_OH)_2_, molecular weight 62.07 (g/mol), and density 1.11 (g/cm^3^) was purchased from Sigma Aldrich, Subang Jaya, Malaysia.

### 2.2. Methods

#### 2.2.1. Material Characterization Techniques

In order to reaffirm the morphology and size of MoS_2_ nanoparticles as per supplier information, field emission scanning electron microscopy (FESEM, JEOL JSM-7600F, operated at 10 kV by JEOL Ltd., Tokyo, Japan) and high-resolution transmission electron microscopy (HRTEM, JEOL JEM-2100F by JEOL Ltd., Tokyo, Japan) were employed, respectively.

#### 2.2.2. Nanofluids Preparation

The samples were prepared with a two-step method in a range of volume fractions (0.005–0.01%) without using any surfactant. The mass of dry powder was calculated to be 0.253 mg/mL, 0.3795 mg/mL, and 0.506 mg/mL corresponding to the volume fraction of 0.005%, 0.0075%, and 0.01%, respectively. The mass of MoS_2_ corresponding to the known volume concentrations was computed using Equation (1).

(1) $$\emptyset =\left[\frac{{\left(\frac{w}{\rho }\right)}_{MoS2}}{{\left(\frac{w}{\rho }\right)}_{MoS2}+{\left(\frac{w}{\rho }\right)}_{EG}}\right]$$

**Figure 1:**
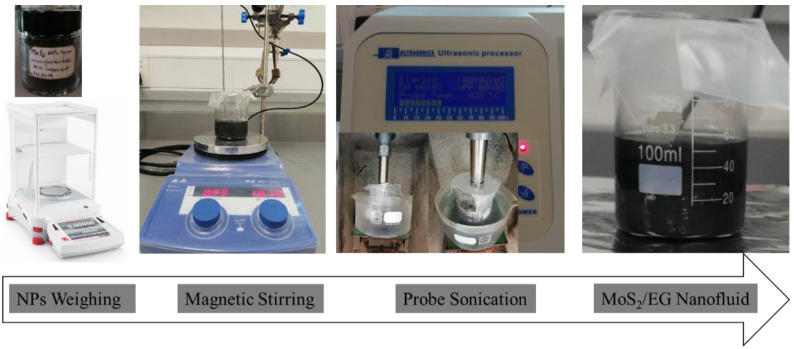
Preparation steps of MoS_2_/EG nanofluids.

The measured dry powder with laboratory balance (Explorer series semi-micro, ±0.0001 g) was mixed with ethylene glycol (EG) and subjected to rigorous magnetic stirring for 30 min maintaining 1010 rpm at an ambient temperature. Subsequently, the de-agglomerate suspension was homogenized with probe-sonication (FS-1200N Ultrasonic Processor, 1200 watt, 20 kHz) at 40% rated power for 30 min. During homogenization, the nanofluids were kept inside an ice bath to prevent excess heating. In order to record true rheological behavior, the obtained nanofluids were tested immediately after preparation. The nanofluids preparation scheme is shown in Figure 1.

#### 2.2.3. Rheological Measurements of Nanofluids

The rheological properties of nanofluids were measured with a dwell time of 10 min to minimize the effects of pre-shear [16,28]. The measurements were carried out with a strain/stress-controlled rheometer (MCR-302, Anton Paar, Graz Austria) equipped with a Peltier cooling system and a double gap measuring geometry (DG26.7-SN61536, 3 mL sample capacity) made from stainless steel. All the rheological measurements were recorded in a temperature range of 0–70 °C with triplicate readings except for shear flow behavior (six measurements), and an average was employed in the analysis. Moreover, detailed information on the rheometer calibrations was provided in our previous study [28].

##### Shear Flow and Temperature Sweep Measurements

The ethylene glycol (EG) has a freezing point ~−12.9 °C and boiling point ~197.3 °C; therefore, the steady-state temperature was used to compute the shear flow behavior in a range of shear rate (0.1–1000 s^−1^) and at various targeted temperatures (0–70 °C). In the heating mode of the temperature sweep test, the test was performed with a pre-shear (50 s^−1^) state for 30 s, followed by a logarithmic temperature ramp @1 °C/min. The cyclic (heating/cooling) temperature sweep (TS) test within 0–50 °C was performed with a pre-shear (100 s^−1^) state for 30 s followed by a logarithmic temperature ramp of 0.5 °C/min. It should be noted that in the cyclic temperature sweep measurements, the cooling phase temperature only reached 0.3 °C. In an optimization attempt, in the case of base fluid, the heating cycles were completed in 60 min (@1.25 °C/min), whereas the cooling cycles could not be completed (back to 0 °C) at the same cooling rate due to the cooling rate limitations of the measuring system. In this particular trial, during the cooling cycle, the temperature could only reach 5.9 °C. To ameliorate it, the heating rate was maintained at @1.25 °C/min while the cooling time was enhanced to 70 min (@1.07 °C/min). Doing so did not provide much relief as the cooling cycle reached 4.64 °C. Here, it is noteworthy to understand that beyond 60 min of time, there was not much significant impact on the cooling rate. Increasing 10 min on top of 60 min did not allow it to cool down the sample holding chamber, which meant more relaxation time was needed to dissipate the heat energy produced during the heating cycle. Successively, heating time was swapped to 80 min (@0.94 °C/min) and concurrently cooling time was enhanced to 100 min (@0.75 °C/min). In this way, the minimum temperature appeared to be around 2.08 °C during the cooling phase. The last trial was conducted on the base fluid by keeping the heating rate at @0.94 °C/min and the cooling time allowed at 110 min, such that the minimum temperature during the cooling phase achieved around 1.82 °C. All these trials on the base fluid suggested that rigorous optimization on experimental conditions is required to study the heating/cooling cycle flow behavior of nanofluids. In view of the base fluid cyclic rheology results and considering the capability of the measuring system, in the case of nanofluid, the heating/cooling analysis was remained limited to a range of temperatures between 0 and 50 °C. Thus, for the case of NF1 (selected for cycling rheology), both the heating and cooling rates remained similar (@0.5 °C/min), such that during the cooling phase, the temperature obtained was around 0.3 °C.

##### Viscoelastic Measurements

To estimate the extent of viscoelasticity, the amplitude sweep (strain sweep) test was performed by varying the strain (0.01–1000%), keeping oscillation frequency (1 Hz) constant. The shear flow behavior between 0.1 and 1000 s^−1^ was studied with a controlled shear rate (CSR) method on a logarithmic scale.

##### Thixotropic Measurements

**Figure 2:**
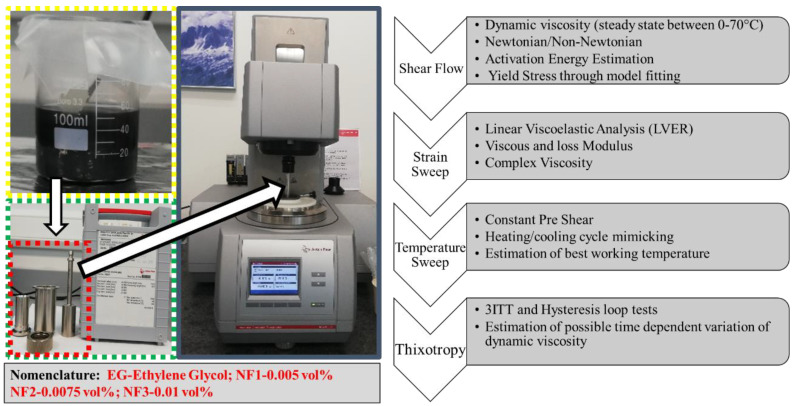
Rheological measurements scheme and nomenclature.

Time-dependent viscosity (thixotropic) was evaluated with a three interval thixotropic test (3ITT) using an O-R-O (oscillation-rotation-oscillation) arrangement: first, the (oscillation) interval determined the static viscosity (low shear rate) in 60 s; second, the (rotational) interval represented the structure break down (high shear rate, 1000 s^−1^) in 60 s; third, the (oscillation) interval corresponded to the structure recovery (low shear as in interval 1) in 500 s. In the 3ITT test, the first and third interval of the strain was computed from the strain oscillation test within the LVER region. Moreover, the hysteresis loop test was also conducted to reaffirm possible thixotropic behavior with an upward curve (0.1–300 s^−1^ in 300 s), a dwell period (60 s at 300 s^−1^), and a downward curve (300–0.1 s^−1^ in 300 s). All the rheological output data analysis was carried out using the computer-controlled RheoCompass software provided by Anton Par. Further details on the calibration of MCR302 were provided in a recent report by our research group [28]. The detailed scheme of rheological measurements and analysis parameters along with nomenclature followed throughout the manuscript is provided in Figure 2.

#### 2.2.4. Thermal Conductivity Measurements

The thermal conductivity of MoS_2_/EG nanofluids was measured using the KD2 thermal property analyzer between 25 and 50 °C. The details on the measuring procedure and calibration were already reported in our previous work [28].

#### 2.2.5. Statistical Analysis

In order to check the statistical significance of steady-state (temperature) viscosity and thermal conductivity, the Student’s *t*-test was employed. The number of measurements (n) was taken as 6 and 10 for viscosity and thermal conductivity, respectively. The data was checked against the null hypothesis for the mean such that there was no difference between the mean of the base fluid and nanofluids. In order to reject the null hypothesis, the test results were obtained in terms of the probability value (*p* < 0.05) and t-stats beyond the critical value within the rejection zone. Further details on statistical analysis were provided in our previous study [29].

## 3. Results

### 3.1. Material Characterization

**Figure 3:**
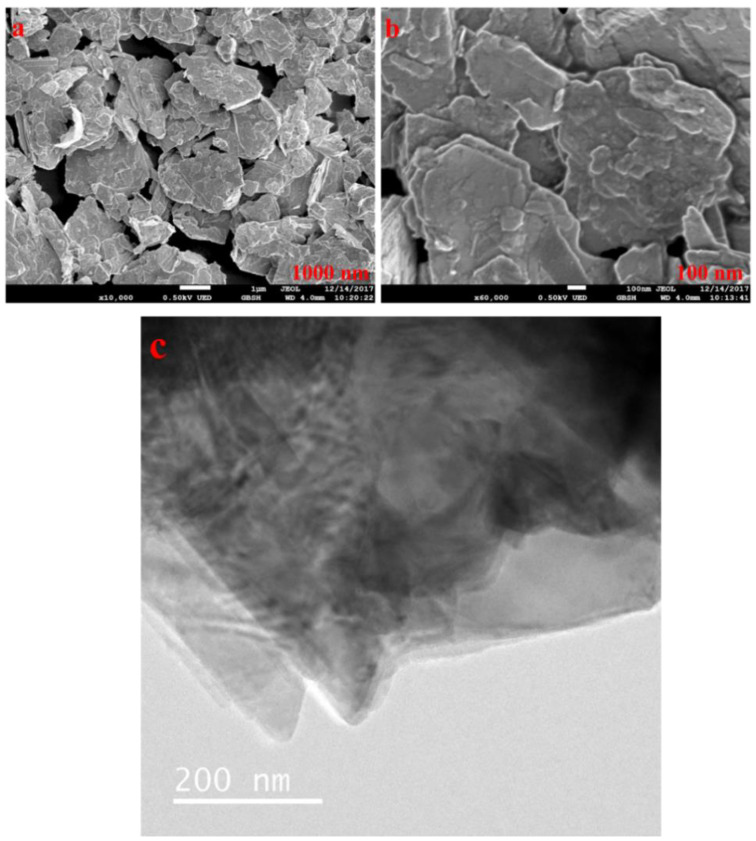
(**a**,**b**) FESEM image of MoS_2_ at different magnifications and (**c**) HRTEM.

Figure 3 show that the MoS_2_ is in the stacked formation with plate/sheet-like morphology, and the size is in compliance with the supplier datasheet.

### 3.2. Rheological Analysis

As most heat transfer systems operate in a range of flow regimes such as laminar and turbulent, the studied shear rates can accurately mimic the viscosity of the nanofluids in these flow regimes. Thus in the present work, to evaluate the flow behavior of the base fluid (ethylene glycol) and nanofluids, the control shear rate (CSR) method was followed because controlling the motor parameters is more accurate compared to the control sample. According to this method, the shear rate (predictor variable) is varied, and the corresponding flow resistance (shear stress) of fluid is measured as a response variable.

#### 3.2.1. Steady-State (Temperature) Shear Flow Behavior Analysis

In order to compare the dynamic viscosity results between temperature ranges from 0 °C to 70 °C, 30 experimental data points were fitted using Equation (4) between the shear rate, 10–1000 s^−1^, for the flow curves. The opted range of shear rate holds well for the Newtonian domain for all the tested nanofluids. Besides the six measurements, the results of shear flow behavior were fitted to determine the Newtonian viscosity, and an average was employed for analysis and comparison purposes. The maximum uncertainty obtained over the entire scale of targeted temperatures was around 5.116%, as shown in Table 1.

Furthermore, to ascertain the statistical significance of shear flow data, a Student’s *t*-test for the mean was conducted, and the reliability of the data was checked through statistical parameters. Mostly, the measurement results appear statistically with a probability *p*-value less than 0.05. The detailed information on statistical analysis is given in Table 2.

**Figure 4:**
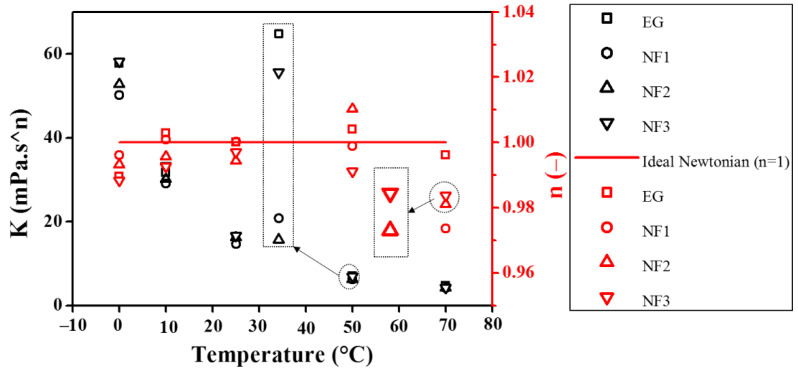
Consistency index (*K*) and flow index (n) obtained from Herschel–Bulkley model fitting.

The Herschel–Bullkley (H-B) Equation (2) and Bingham plastic Equation (3) were also examined within 10–1000 s^−1^ to determine the yield stress $\left(\tau_{o}\right)$, consistency (*K*), and flow index (n). The consistency index (*K*) and flow index (n), obtained using Equation (2), are presented in Figure 4.

(2) $$\tau =\tau_{o}+K{\dot{\gamma }}^{n}$$

(3) $$\tau =\tau_{o}+\mu \dot{\gamma }$$

It can be seen from Figure 4 that the flow index (n) is pretty close to the unity, which means Equation (4) can be employed with confidence to compute the dynamic viscosity from the experimental viscosity curves within 10–1000 s^−1^. It should be noted that the maximum uncertainty obtained over the entire temperature scale was ~5.116% (Table 1). Therefore, taking into account this uncertainty, the value of n can be marginalized to 0.95 instead of 1 when referring to the behavior of the fluid as Newtonian. Interestingly, in the present work, the H–B model shows the n value between 0.97375 and 1. This variation can occur as a result of rheometer accuracy, whilst the consistency index (*K*) also appears to be in great agreement with the temperature-dependent shear flow curves as *K* decreased with increasing the temperature. In addition, the *K* values are also good indicators for the apparent dynamic viscosity. It can be seen from the *K* values that the NF1 has less value than EG, showing better flow behavior, whereas with the increased concentration, it almost approaches the EG value. Recently, Yadav et al. described that EG behaves non-Newtonian in a shear rate range between 0 and 150 s^−1^. It should be noted that EG appeared Newtonian beyond 10 s^−1^ in the present work, which means the measurements are highly dependent on the measuring device sensitivity. In addition, their work regraded EG as non-Newtonian in the temperature range of 30–50 °C, whereas it was regarded as Newtonian in the range of 70–90 °C, which is inconsistent with the present results [27]. Moreover, the detailed fitting parameters obtained using Equations (2) and (3) are shown in Table 3. Generally, the yield stress value should increase with the concentration of particle loadings while it should decrease with temperature. However, the sensitivity of the measuring system (rheometer) does matter a lot when following the general trend.

#### 3.2.2. Influence of Steady State Temperature and Concentration on Viscosity

**Figure 5:**
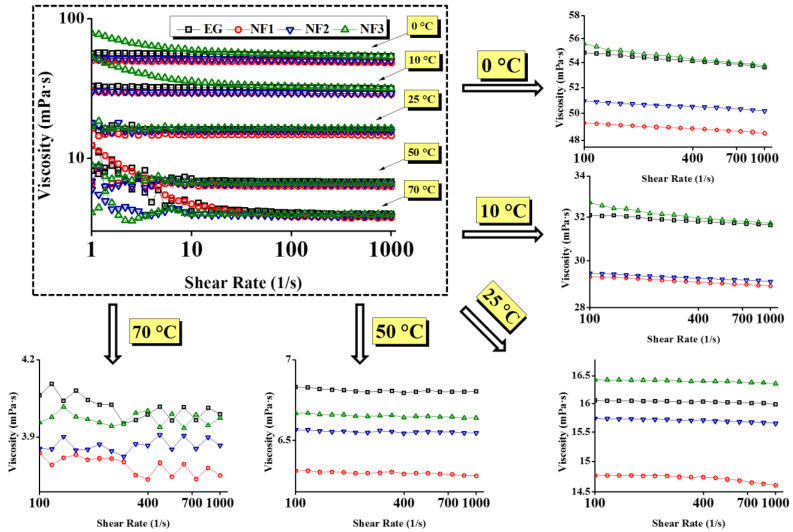
Apparent dynamic viscosity curves as a function of shear rate at various concentrations (NF1—0.005%; NF2—0.0075%, and NF3—0.01%) and steady-state temperatures (0–70 °C).

**Figure 6:**
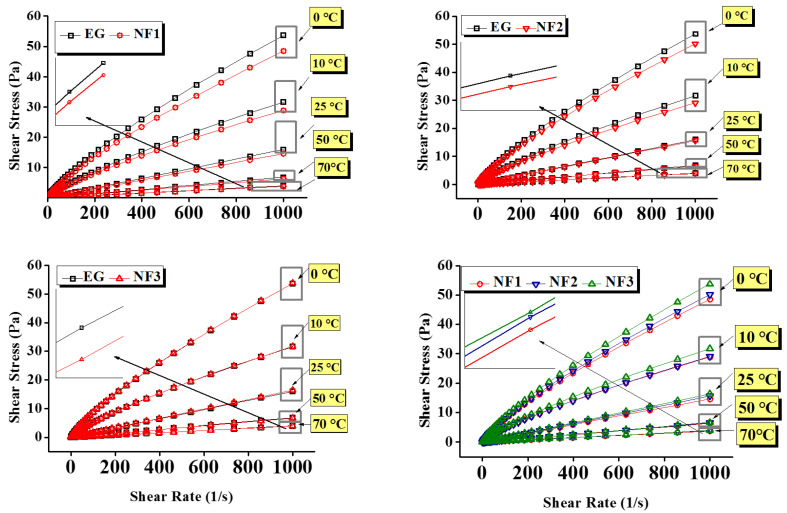
Flow curves of nanofluids and EG as a function of shear rate over various concentrations (NF1—0.005%; NF2—0.0075%, and NF3—0.01%) and steady-state temperatures (0–70 °C).

Further, the viscosity curves and flow curves are shown in Figure 5 and Figure 6, respectively. Figure 6 represent that in between 10 and 1000 s^−1^, the shear stress increases linearly with the shear rate, and the same trend follows at all targeted temperatures with the exception of 0 °C and 10 °C. Additionally, taking into account the flow index obtained from the H–B model, the results can be modelled using Newton’s law of shear stress as follows:

(4) $$\tau =\mu .\dot{\gamma }$$

In the above equation, parameters τ, μ, and $\dot{\gamma }$ represent shear stress, dynamic viscosity, and shear rate, respectively.

The comparative steady state (temperature) viscosity of nanofluids (NF1, NF2, and NF3) w.r.t EG is depicted in Figure 5 over a range of shear rates. At low shear rates up to 10 s^−1^, the pure EG shows Newtonian behavior up to 10 °C. Such behavior is obvious as, at low temperatures, the molecules are less excited and remain intact. Therefore, the torque values are sufficient to detect the flow resistance offered by the EG. Likewise to EG, NF1, and NF2 also retain the same behavior; however, NF3 response become clouded. As NF3 appear to have shear thinning behavior, therefore it can represent an optimum concentration to control the flow behavior of nanofluids such that it remains similar to EG. The reason behind such clouded behavior lies within the modified structure of NF3, which could form the intact clusters. Therefore, it may offer more resistance on top of EG, as indicated in Figure 5. Furthermore, below 10 s^−1^, the increasing temperature beyond 10 °C gives rise to Brownian movement, which can be observed between 25 and 70 °C in ascending. This results in oscillatory behavior of viscosity; in this case, the torque sensed by the measuring system fluctuates due to randomly oriented nanosheets. Subsequently, when the shear rate sweeps from 10 to 1000 s^−1^, the nanosheets align in the flow direction resulting in the fairly constant viscosity for all samples with the exception of NF3. As in the case of NF3, the viscosity continues to decline due to its cloudy behavior up to 10 °C. Beyond that, it follows the same trend as others due to decreased cohesive forces.

Here, it is essential to highlight that the addition of MoS_2_ significantly reduces the viscosity of nanofluids. For instance, it is obvious from Figure 5 that the viscosity of nanofluids remained below pure EG. Concurrently, the shear stress values also show remarkable reduction as compared to EG, which means low input is needed to maintain the same flow conditions (Figure 6). However, the temperature and concentration become influential variables that can alter the flow behavior of nanofluids. At a temperature of 0 °C, the maximum viscosity reduction was observed for NF1, which began to increase when the concentration increased (NF2, NF3). Because of NF3, the viscosity curve almost coincided with the EG. With a subsequent temperature rise to 10 °C, the reduction behavior persisted, but the viscosity curve corresponding to NF2 appeared slightly above the NF1. Continuing with temperature rise, surprising behavior was observed between 25 and 70 °C, as the NF3 viscosity curve appeared below EG, whereas the NF1 remained at the bottom with a tangible margin as compared to NF2 and NF3.

It is well known that the temperature rise may cause dispersion instabilities leading to the sedimentation process [16]. Therefore, rheological studies should be preferred up to the lowest possible temperature range. This is witnessed in Figure 5 at 70 °C, where the fluctuations in measurements become significant. That is why in the present work, any subsequent temperature was not explored.

**Figure 7:**
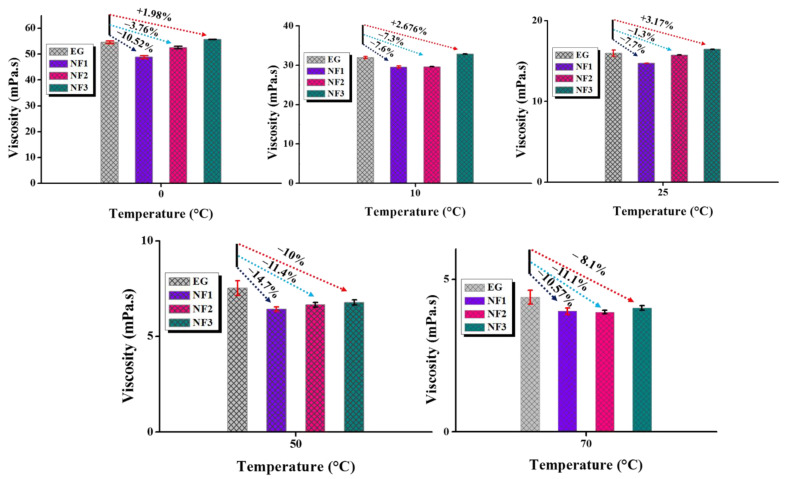
Calculated values of dynamic viscosity using Newton shear stress law and comparison with ethylene glycol (EG) at various steady-state temperatures.

Based on Equation (4), the average dynamic viscosity values with uncertainty (@95% confidence interval) are shown in Figure 7. It is apparent that the steady-state (temperature) viscosity for NF1 is fairly below EG with a maximum reduction of ~14.7% at an operating temperature of 50 °C. However, NF2 and NF3 also become the active candidates with increasing temperature as they also show viscosity reduction but overall less than NF1. A summary of viscosity reduction and enhancement is provided in Table 4.

**Figure 8:**
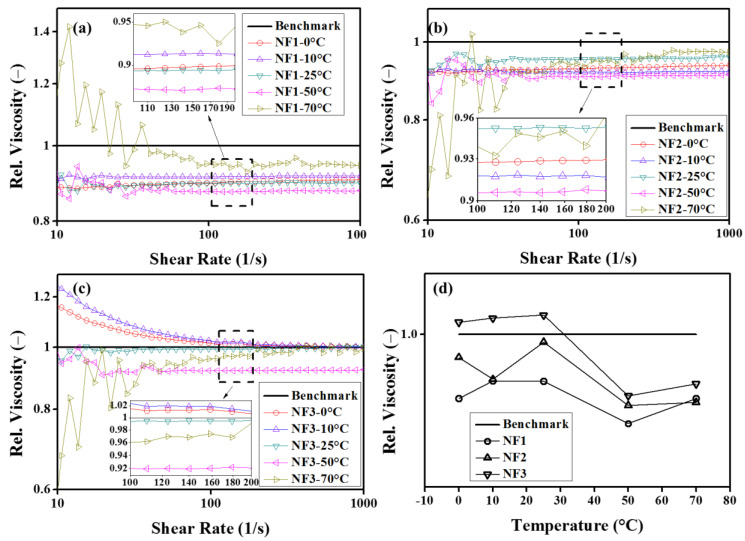
Relative viscosity as a function of shear rate (**a**–**c**) and steady-state temperature (**d**).

In order to observe the behavior of relative viscosity over a range of shear rates and steady-state temperatures, the results are given in Figure 8. It can be seen from Figure 8a,b that the relative viscosity is independent of the shear rates, particularly for NF1 and NF2 up to 50 °C. At 70 °C, the oscillatory behavior is ascribed to the dispersion instabilities. However, NF3 show the same trend as observed in the viscosity curves, as shown in Figure 8c. In addition, from these observations of steady-state viscosity, the best working temperature of nanofluids appears around 50 °C, as depicted in Figure 8d.

#### 3.2.3. Arrhenius Plot and Activation Energy from Steady State Viscosity Data

Furthermore, the steady-state calculated dynamic viscosity corresponding to all targeted temperatures, as shown in Figure 7, was fitted with the Arrhenius Equation (5).

(5) $$\eta =\eta_{\infty }.exp^{\frac{E_{a}}{RT}}$$

(6) $$\ln\left(\eta \right)=\ln(\eta_{\infty })+\left(\frac{E_{a}}{R}\right)\left(\frac{1}{T}\right)$$

The parameters in the above equations such as *η*, *η*_∞_, *E_a_*, *R* and *T*, correspond to the experimental viscosity at a particular given temperature, viscosity at an infinite temperature, activation energy, universal gas constant (8.314 JK^−1^ mol^−1^), and operating temperature, respectively. It should be noted that there is a non-negative *E_a_* value in the above equations because it is the derivative form in terms of viscosity rather than the actual rate form [30].

**Figure 9:**
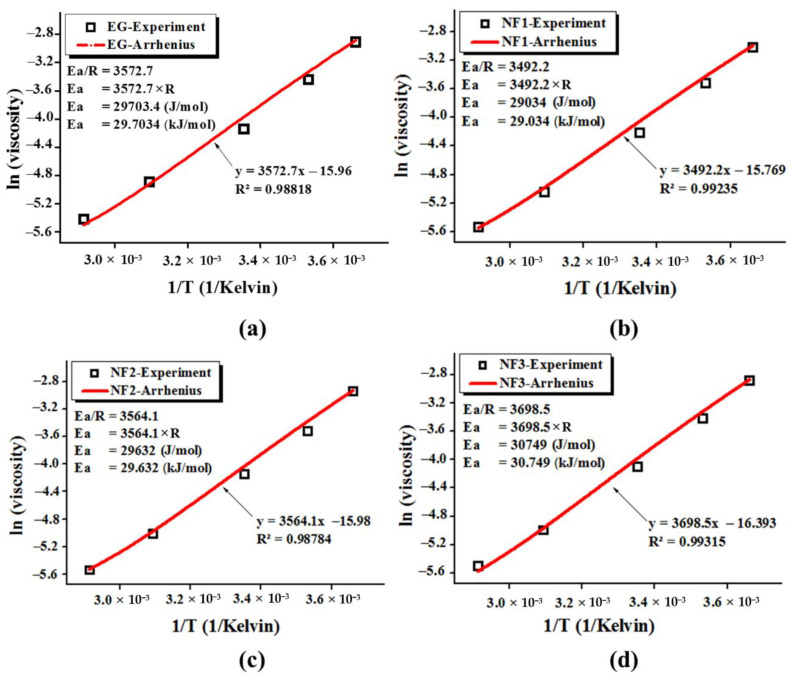
Relationship between the experimental and Arrhenius Equation (6) fitting for activation energy (Ea) estimation where viscosity is in Pa·s while the temperature is in Kelvin; (**a**) EG, (**b**) NF1, (**c**) NF2 and (**d**) NF3.

The calculated data from the experimental shear flow behavior fitted well with Equation (6) as the coefficient of determination (R^2^) appeared in a range of 0.98784–0.99315. The Arrhenius Equation (5) fitting parameters, along with the graphical representation, are shown in Figure 9.

The tailored activation energy of ethylene glycol with MoS_2_ dispersion can explain the variation in the viscosity values as it is obvious from Figure 9 that the calculated Ea for ethylene glycol is close to the literature value of 29.703 kJ/mol. Further, with the addition of MoS_2,_ the Ea value reduced to 29.034 kJ/mol, 29.632 kJ/mol for NF1 and NF2, whilst it increased to 30.749 kJ/mol corresponding to NF3. This means that with an increasing concentration of MoS_2,_ the cluster formation becomes intact and intense, which may enhance the viscosity of nanofluids. The expected reason behind the reduced Ea value may be ascribed to the bond perturbations, which can also reduce the viscosity [31].

#### 3.2.4. Temperature Sweep Heating/Cooling Cycle Analysis

Hitherto, there is no study found in the literature which emphasizes the heating/cooling behavior of the MoS_2_/EG nanofluids from low to high temperatures. Mostly, the research studies focusing on the convective heat transport benefits through nanofluids deployment do not consider this particular aspect pertinent to cyclic rheology. Rather, the single viscosity values are computed either through the use of viscometer/rheometer, which could be misleading when being used in analytical relations to calculate the desired properties of the heat transfer systems. As in the heat transfer systems, the working fluid (nanofluid) undergoes consecutive heating and cooling cycles which can significantly influence the system’s performance if its flow properties are not explored in such manners. Further, the knowledge of the exact flow behavior of nanofluids can also assist computation researchers in predicting the properties of nanofluids with a close proxy. Therefore, in order to simulate the heating/cooling cycle behavior of MoS_2_/EG nanofluids, the optimized sample (NF1) from the shear flow behavior analysis was selected.

**Figure 10:**
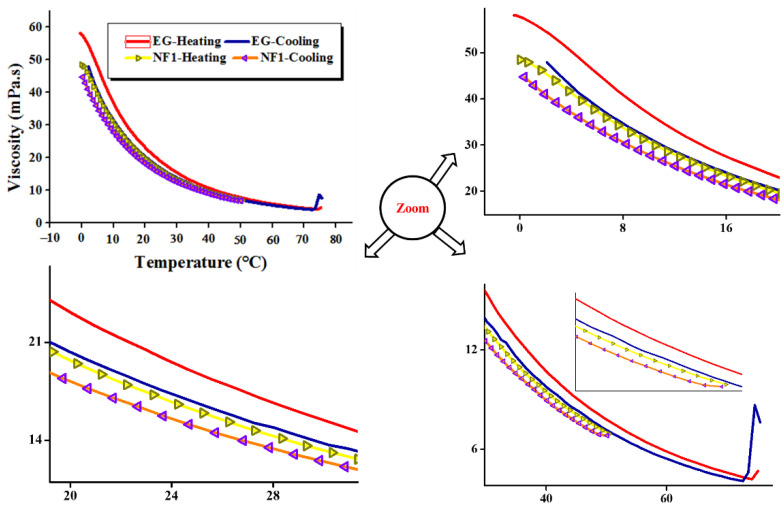
Cycling dynamic viscosity to simulate the true flow behavior of MoS_2_/EG nanofluids in consecutive heating and cooling cycles at a shear rate of 100 s^−1^.

To achieve the desired cyclic behavior of nanofluids, firstly, the base fluid (EG) was simulated between 0 and 75 °C for the reference curves as well as to optimize the heating and cooling time of the samples. As given in Figure 10, the 100 s^−1^ pre-shear rate was considered for the temperature sweep analysis both in the heating and cooling cycles. Figure 10 show a clear hysteresis loop in the heating and cooling cycles. The heating curve remains on top of the cooling, both for EG and NF1. Interestingly, the NF1 shows a viscosity curve below EG during heating and cooling cycles. This means that in order to maintain the same flow even in temperature ramp, low input energy is required in the case of NF1 as compared to EG.

**Figure 11:**
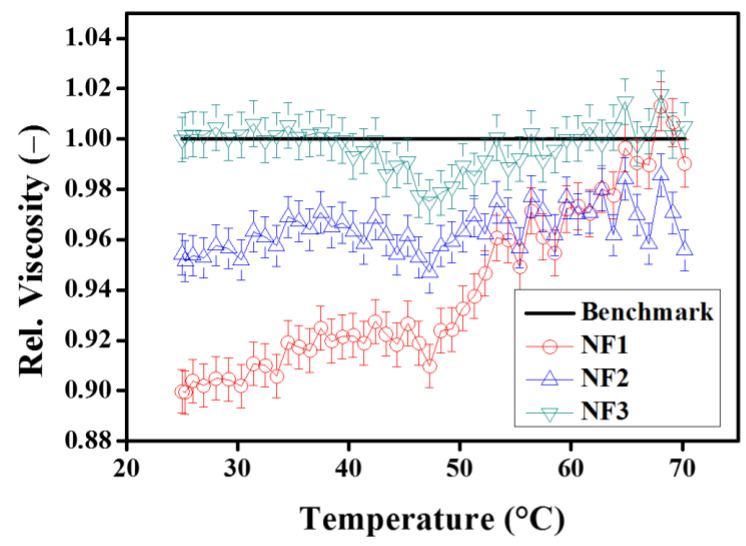
Relative viscosity as a function of temperature ramp in heating mode (25–70 °C) with pre-shear (50 s^−1^).

Figure 11 show the variations in relative viscosity during temperature ramp in heating mode. As it is evident from the figure, the relative viscosity increases with increasing temperature. This means that at higher temperatures, the viscosity of the base fluid decreases faster as compared to the nanofluids. This may be due to the enhanced Brownian motion at higher temperatures which may intensify the inter-particle collisions. However, interestingly, the minimum relative viscosity trend remained prominent around 50 °C as already observed in the steady-state temperature response for NF1 (Figure 8d).

In short, cyclic rheology is a good tool to simulate the flow behavior of nano coolants (nanofluids) before their deployment into applications. This could help to assess the effective number of consecutive heating/cooling cycles that one fluid is supposed to perform effectively in continuous operation. Thus the entailing characteristics of nanofluids as a result of cyclic rheology should be considered essential for future characterization work pertinent to liquid nano coolants.

#### 3.2.5. Viscosity Enhancement and Reduction Mechanisms

It was reported that the shear-thinning behavior of nanofluids occurs as a result of nanoparticles arrangement/network. Nanoparticle alignment is probably induced in the flow direction as the shear rate is increased. In some cases, the colloids may be unstable under shearing since black aggregates of nanofluids were observed at the centre of the rheology cell. These aggregates probably contain a large amount of the initial nanoparticles, which impoverishes the solution in dispersed nanofluids, and the rheological behavior of the NFs is consequently mainly governed by that of the solvent [32]. It was noted that the small concentration and nano-size particles did not affect the viscosity of the base fluid. Only ~4% in increment was seen as compared to the base fluid, which was ascribed to less concentration and small size, leading to reduced friction among the base fluid layers [33]. Moreover, at low shear rates, nanofluid entanglement due to high surface energy caused more resistance to flow, which subsequently reduced as the shear rate increased as the particle size was broken into small units. Consequently, the Nanofluids arranged themselves in the flow direction, and as a result, viscosity showed reduction compared to the low shear value [34]. In another report, it was also shown that the fluid molecules arrangement momentarily altered as the spindle revolved at low shear rates. Additionally, progressively, the fluid molecules lined up in the increasing shear orientation, causing less opposition and a decrease in viscosity. Nevertheless, at a very high shear rate, the maximum possible shear organization was achieved. The bigger clusters were fragmented down to minor sizes, subsequently causing small friction and consequently small viscidness [35].

In addition, the maximum mitigation in viscosity (~73–75%) was evidenced as the temperature sweeped from 40 to 100 °C. This was attributed to enhanced Brownian motion among the nanoparticles as the temperature resulted in weak van der Waals forces acting on nanoparticles [36]. Furthermore, molecular dynamic (MD) simulation showed that the improved hydrophilicity might also reduce the viscosity [37]. Another MD study revealed that the viscosity of the NFs decreased with increasing nanofluids size while aggregation caused an increase in viscosity [38]. Thus the understanding of the temperature-dependent viscosity of nanofluids is also essential when dealing with elevated operational temperatures. Mainly, viscosity is related to the interatomic bonding strength among the molecules of nanofluids. The bonding strength is governed by many factors such as molecular structure, shape, and kinetic energy, which is directly linked with nanofluids system temperature. As a result of the temperature rise, the bonding weakened along with high kinetic energy leading to reduced viscosity. It was reported the temperature rise of base fluid (pure water) showed a significant reduction in viscosity up to 50%. At the same time, nanofluids showed a ~20–30% reduction at same working conditions. Therefore, it was postulated in the case of nanofluids, that less viscosity reduction with temperate was attributed to the fact the nanofluids absorbed thermal energy [39]. In many reports, researchers adopted surfactants as stabilizers in NF production. With temperature rise, viscosity showed an increment in the presence of PVP surfactant for aqueous-based nanofluids. It was tributed to excess counter ions of PVP surfactant, which promoted the growth of micelles leading to additional resistance to flow. It was noteworthy that such a phenomenon was not noted for EG-based nanofluids. Probably, for EG-based nanofluids at high temperatures, the hydrogen bonding broke and the van der Waal forces became less, which showed decreasing viscosity. The increase in temperature might detach–OH functionalities attached to MWCNT, which could be regarded as another reduction mechanism attributed to the rise in temperature. These results suggested the suitability of PVP surfactant for EG compared to DIW [40]. Therefore, the modification in colloidal suspension ionic strength also affects the viscosity. The addition of electrolytes alters the net surface charges on nanoparticles. This thrusts the change in the fractal dimension of aggregates with surface topography. On the contrary, any variation in ionic strength around the small size particles in nanofluids affected the electrical double layer. In this case, the primary electro viscous coefficient (p) played a vital role in deciding the rheology. *p* primarily depends on the solid–liquid interface electrical state. Consequently, the adjustment of NF’s pH significantly alters the fractal dimension and *p*-value which can cause variation in viscosity [41].

Besides, some studies also highlighted the potential of low dimension materials and surfactants in decreasing the viscosity. Such reduction could be either due to the low concentration or self-lubrication characteristics of the dispersed phase. Baheshti et al. showed a ~0.9% reduction in viscosity for transformer oil-nanotubes nanofluids at 0.01 wt.% [42]. It was shown the two dimensional (2D) sheets, such as the morphology of nanoparticles, might stimulate co-particle sliding behavior leading to the self-lubrication phenomenon. This could be assigned to the oleophilic behavior of 2D sheets [43]. Anti-friction materials also showed better lubrication characteristics. The reduction in BN/EG nanofluids viscosity at 1 wt.% PVP concentration was reported [44]. The viscosity of the nanofluids decreased with increasing the size of the particle. It was described, at the same concentration of nanoparticles, for small size particles, a greater number of interactions sites developed with base fluid, which increased the contact surface area leading to a more viscous nature [45]. Another report urged that the graphene/oil nanofluids reduced viscosity which was attributed to the self-lubrication of graphene created by the exclusive 2D planar structure. However, the higher concentration of nanoparticles might cause an increment in viscosity due to the large resistance imparted by a large number of nanoparticles. A maximum reduction in kinematic viscosity of ~12% was noted [46]. Recently, Zhou et al. stated the unprecedented behavior of aqueous-based TiO_2_ NFs. It was shown that the addition of SDS surfactant TiO_2_ nanofluids significantly reduces the viscosity of water when added individually. Furthermore, this reduction in viscosity was also noted as a synergistic effect when added collectively inside the water. The reduction in dynamic viscosity with SDS and nanofluids was attributed to the nanofluids lubrication effect and analogous micelle formation [47]. Similar observations were also reported by shah et al. for the rGO/EG based nanofluids [28]. Moreover, it was described that the surfactants added into liquids became adsorbed onto solid surfaces and caused less friction [48]. More recently, ceria/EG nanofluids showed ~33% viscosity reduction along with a critical temperature limit of ~65 °C. Beyond such a limit, the viscosity starts to increase. It assumed that the shear-induced particles migration might have caused this reduced viscosity due to the non-uniform viscosity field along the transverse plane [27].

#### 3.2.6. Strain Sweep Analysis

**Figure 12:**
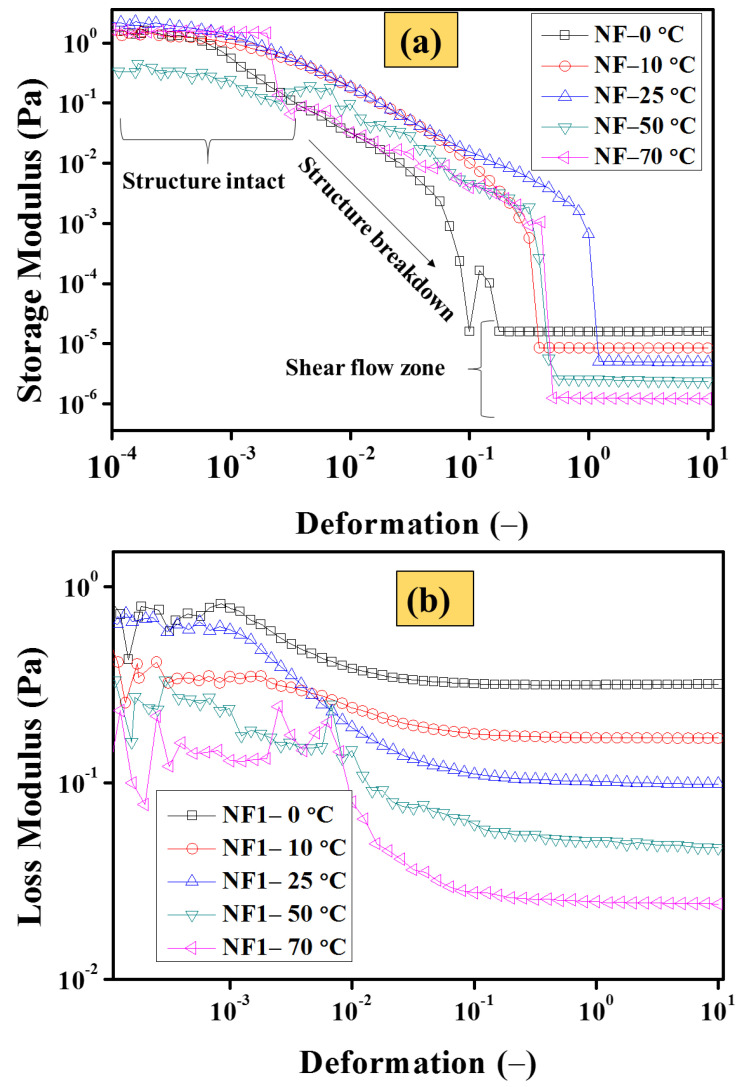
Oscillatory strain sweep analysis at a constant frequency of 1 Hz for NF1 (**a**) storage (G′) and (**b**) loss (G″) modulus.

As shown earlier in Table 4, the consolidated viscosity results reveal that NF1 (0.005 vol%) can be considered the optimum nanofluid. Therefore, in order to further elucidate the structural changes with particle loading, NF1 was studied using an oscillatory strain sweep test, as shown in Figure 12. It is apparent from Figure 12a that the MoS_2_ addition induces structure within the base fluid. At low deformation (oscillatory disturbance caused by measuring geometry), the structure remains intact at all targeted temperatures with sufficient G′ values, which vary with temperature. Interestingly, at 50 °C, the G′ appear below other targeted temperatures, which means that at this particular temperature, the structural re-orientation starts at 70 °C and the Gʹ becomes large compared to that at 50 °C. Such behavior is an indication that temperature significantly introduces the re-arrangement of colloids. This could be a plausible reason behind the increasing trend of relative viscosity, as shown earlier in Figure 11. Furthermore, with increasing deformation, the structure break starts and keeps on going until the interaction becomes weak between the MoS_2_ sheets. At this point, all the Gʹ curves are aligned and appear well in agreement to the shear flow behavior as stated earlier. This is appealing that once the internal structure of NF1 completely broken, the G′ decreases with increasing temperature. Similar behavior is also followed by the G″, as when the temperature increases progressively, the viscous dissipation reduces due to the weak interaction between EG molecules.

Here, one can establish an interesting analogy between the dynamic viscosity from shear flow analysis and complex viscosity $\left(\eta^{*}\right)$ between shear rates beyond 1 s^−1^. According to the Cox–Merz rule, the $\eta^{*}$ can be written as follows [49]:

(7) $$\eta^{*}=\frac{G^{*}}{\omega }$$

In Equation (7), the $G^{*}$ and ω are complex modulus (Pa) and oscillation frequency (rad/s), respectively. However, in the simplest approach, the complex viscosity is computed using Equations (8) and (9).

(8) $$\dot{\gamma }=⍵\gamma$$

(9) $$\eta^{*}=\frac{\tau }{\dot{\gamma }}$$

**Figure 13:**
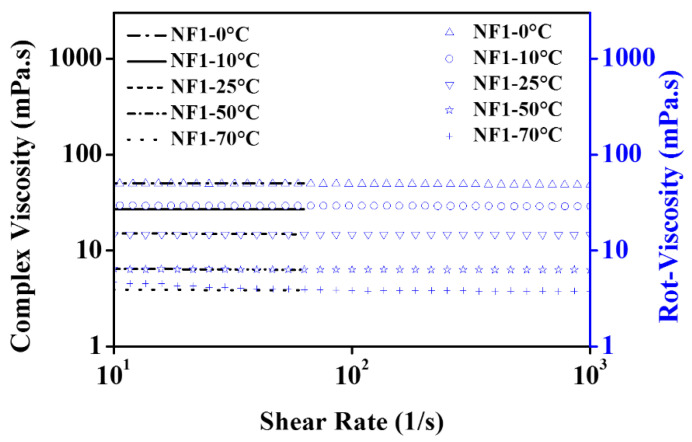
Comparison of complex viscosity and dynamic viscosity from shear flow behavior for NF1.

In Equations (8) and (9), $\dot{\gamma }$, γ, and τ are the shear rate (s^−1^), shear deformation (-), and shear stress (Pa), respectively. Thus in the present work, the $\eta^{*}$ is calculated using Equation (9) and compared with the dynamic viscosity obtained from shear flow analysis within 1–1000 s^−1^. The results are shown in Figure 13. The results clearly show that a plausible analogy between these two tests exists which means there is remarkable potential in MoS_2_/EG nanofluids to tailor the flow characteristics of EG. Therefore, the results of shear flow behavior in rotational rheology can also be checked and verified through oscillatory measurements.

##### ITT and Hysteresis Loop Analysis

In view of better rheological performance by NF1, the time-dependent behavior was studied using 3ITT and hysteresis loop analysis.

**Figure 14:**
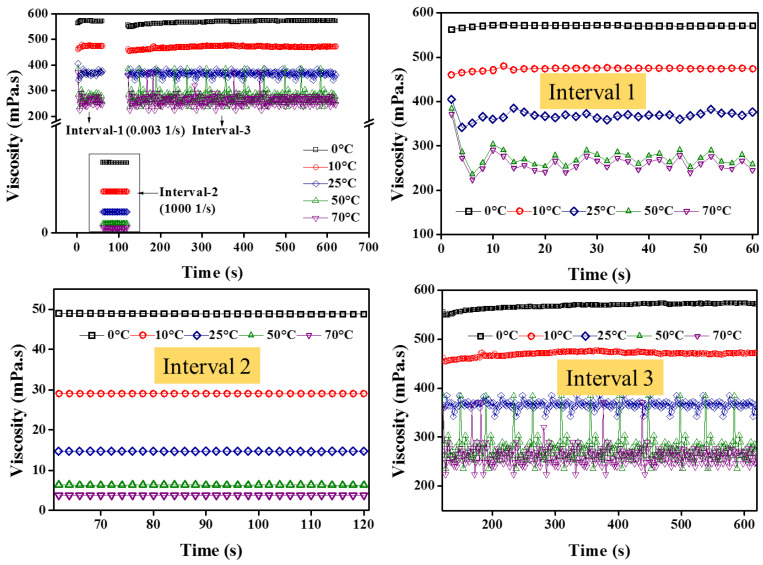
Three intervals thixotropic test (3ITT) for NF1.

**Figure 15:**
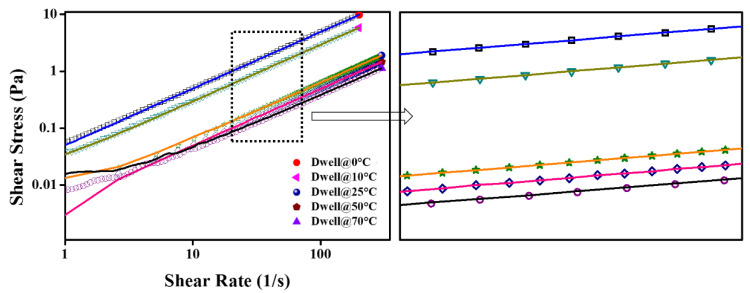
Hysteresis loop test for NF1; the bullets represent an upward curve while solid lines show downward.

It can be observed from Figure 14 that during interval-1, the zero shear viscosity (at low shear rates) is quite high, showing stable colloids. However, with increasing temperature, the reducing trend follows NF1. On the other hand, when the NF1 is completely sheared (interval-2), the dynamic viscosity is similar, as previously observed in the steady-state (temperature) shear flow behavior. More importantly, interval-2 shows quite stable viscosity over test time. Furthermore, upon removing the high shear state in interval-2, the sample was allowed to regain its initial state in interval-3. It is clear from Figure 14 that the NF1 reach back to the initial state in interval-3 without any loss of energy, as applied in interval-1. Such behavior indicates the non-thixotropic response of NF1. Besides, to reaffirm such a non-thixotropic state of NF1, the hysteresis loop results in Figure 15 show no energy loss during the upward and downward curve, particularly beyond 10 s^−1^. Thus based on the consolidated outcomes from 3ITT and hysteresis loop, the NF1 is regarded as non-thixotropic in nature.

### 3.3. Thermal Conductivity

**Figure 16:**
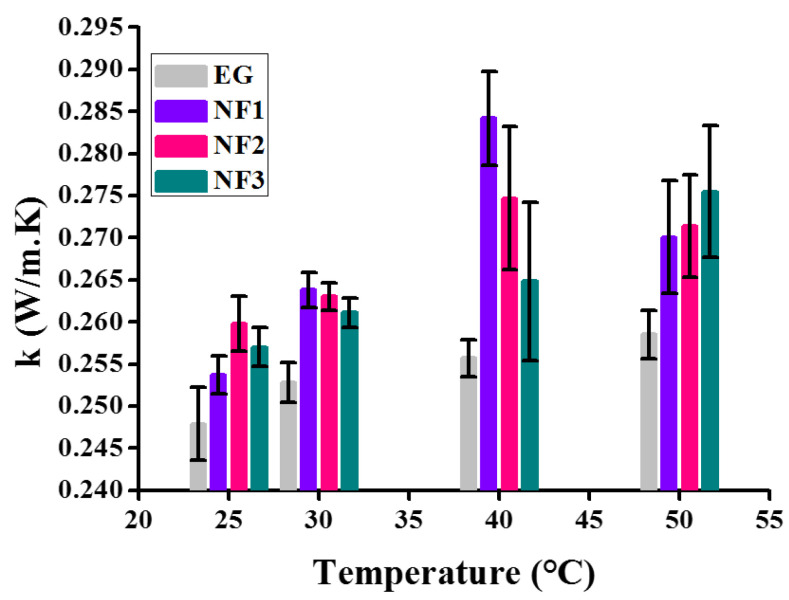
Variation of thermal conductivity over various temperatures.

Generally, it is described as the thermal conductivity increasing with the increasing concentration of colloids. Therefore, in order to observe the complete thermal behavior of all nanofluids (NF1, NF2, and NF3) employed in the present work, it was also computed, as shown in Figure 16.

The thermal conductivity data appear significant using the Student’s *t*-test, as shown in Table 5. For the sake of comparison, the thermal conductivity of EG is also shown. All the data in Figure 16 is presented with error bars corresponding to the absolute average deviation (AAD) in the measurements. The results indicate that up to 40 °C, the thermal conductivity increases with temperature for all nanofluids. However, beyond 40 °C, NF1 and NF2 show a decrement while NF3 appears to increase.

Here, it is noteworthy to mention that such behavior with temperature can be ascribed to the stability of nanofluids over time. At 25 °C, NF2 and NF3 appear to have more thermal conductance, but with temperature, sweep transformation occurs. This is an indication that the colloid’s stability with temperature significantly reduced, resulting in sedimentation over time. Therefore, here, the stability study is out of the scope of present work, which shall be followed in future work. Overall the summary on thermal conductivity is given in Table 6.

## 4. Performance Criteria

The convective heat transport inside a pipe/duct for the turbulent flow conditions is given as follows [27]:

(10) $$h\propto \;\mu^{-0.47}.k^{0.67}$$

In Equation (10), *h,* μ*,* and *k* refer to the convective heat transfer coefficient (W/m^2^·K), dynamic viscosity (Pa·s), and thermal conductivity (W/m·K), respectively. As in the present work, a significant viscosity reduction was observed along with thermal conductivity enhancement. Thus, the use of MoS_2_/EG nanofluids can improve the thermal transport efficiency of convective heat transfer devices.

## 5. Conclusions

Considering the importance of nanofluids in convective heat transport devices, in the present work, MoS_2_/EG nanofluids were studied for their rheological properties in a wide temperature range, 0–70 °C. Morphological analysis through microscopy verified their sheet-like architecture. The shear flow behavior reveals that at a low shear rate, non-Newtonian behavior prevails, whereas the high shear rates beyond 10 s^−1^ transformed the nanofluid’s nature into Newtonian fluid. The shear flow analysis also shows an optimum concentration for MoS_2_/EG nanofluids at which considerable viscosity reduction can be achieved w.r.t EG. Activation energy (Ea) analysis shows that the addition of MoS_2_ within EG causes a reduction in Ea, which varies with concentration. The reduced activation energy probably perturbed the hydrogen bonding, which resulted in the easy shearing of EG layers. Besides, the relative viscosity remained independent over a range of shear rates for steady-state (temperature) viscosity measurements, particularly around 50 °C. Concurrently, the relative viscosity reflects significant variation with temperature, showing that the optimum temperature appears to be around 50 °C. Furthermore, the oscillatory rheology clearly indicates structural networks along with rotational test verifications. Finally, the thermal conductivity analysis shows considerable thermal conductivity enhancement up to ~11%. Therefore, the MoS_2_/EG nanofluids can be employed as a convective heat transport medium with the possibility to improve the remarkable thermal performance of the system.

## Figures and Tables

**Table 1:** Uncertainty data on the dynamic viscosity.

Sample (s)	Temperature (°C)				
	0	10	25	50	70
	Uncertainty on Apparent Viscosity (%)				
EG	0.627	0.621	2.443	5.089	5.116
NF1	1.513	0.909	0.084	1.862	2.625
NF2	0.934	0.193	0.185	1.782	1.438
NF3	0.232	0.250	0.133	2.028	1.888

**Table 2:** Statistical significance on dynamic viscosity measurements using the student *t*-Test for mean at a confidence interval of 95%.

Sample (s)	Temperature (°C)	DoF		
		5		
		*t*-Stat	*p*-Value	Remarks
NF1	0	12.86	5.06 × 10^−5^	Significant
NF2		4.13	0.009008	Significant
NF3		5.62	0.002459	Significant
NF1	10	10.06	0.000166	Significant
NF2		16.77	1.38 × 10^−5^	Significant
NF3		10.75	0.00012	Significant
NF1	25	13.28	4.32 × 10^−5^	Significant
NF2		4.74	0.0051	Significant
NF3		−2.16	0.0831	Not significant
NF1	50	3.87	0.0117	Significant
NF2		2.90	0.0336	Significant
NF3		2.38	0.0627	Not significant
NF1	70	3.68	0.0142	Significant
NF2		3.50	0.0171	Significant
NF3		2.43	0.0593	Not significant

**Table 3:** H–B and Bingham Model fitting parameters.

Sample Description	Temperature	Model Fitting Parameters						
		H-B				Bingham		
	(°C)	τ_0_ (mPa)	K (mPa·s^n^)	n (−)	R^2^	τ_0_ (mPa)	μ (mPa·s)	R^2^
EG	0	−1.2902	57.832	0.98963	0.99999	13.733	55.208	0.99998
NF1		−1.7728	50.273	0.99623	1	3.0693	49.434	1
NF2		−3.6035	52.799	0.9931	1	5.6223	51.193	0.99999
NF3		89.42	58.17	0.98835	1	135.33	53.86	0.99997
EG	10	4.6871	31.746	1.0029	0.99999	0.3406	31.831	1
NF1		2.3201	29.223	1.001	1	1.579	29.351	1
NF2		0.14655	30.128	0.99557	1	3.5528	29.537	1
NF3		81.15	33.28	0.99264	1	98.14	31.7	0.99999
EG	25	3.2682	16.499	1.0001	0.99999	4.8633	16.486	0.99999
NF1		−0.29122	14.761	1.0003	1	−0.40126	14.781	1
NF2		−2.0878	16.148	0.99437	1	0.031177	15.755	1
NF3		−1.3611	16.67	0.99697	1	−0.04554	16.447	1
EG	50	3.6218	7.082	1.0041	0.99998	4.5747	7.2856	0.99994
NF1		1.1426	6.333	0.99904	0.99999	1.2875	6.3063	0.99999
NF2		2.9969	6.246	1.0103	0.99998	1.2447	6.5379	0.99998
NF3		−0.68001	6.9255	0.99116	0.99998	0.73365	6.6623	0.99998
EG	70	0.80308	4.7863	0.99623	0.99993	1.3488	4.7187	0.99993
NF1		7.2659	4.23	0.97375	0.99989	9.759	3.7666	0.99981
NF2		−0.7373	4.2266	0.98109	0.99993	1.0563	3.8898	0.99985
NF3		−1.1814	4.2835	0.98369	0.99994	0.39501	3.9876	0.99989

**Table 4:** Dynamic viscosity enhancement/reduction at various temperatures and concentrations (negative sign indicates reduction compared to base fluid).

Sample Name	Viscosity Enhancement/Reduction [%]				
	0 °C	10 °C	25 °C	50 °C	70 °C
NF1	−10.52	−7.59	−7.70	−14.66	−10.51
NF2	−3.76	−7.34	−1.28	−11.64	−11.14
NF3	1.97	2.67	3.17	−10.05	−8.10

**Table 5:** Student’s *t*-test parameters on thermal conductivity data corresponding to 95% confidence interval.

Sample (s)	Temperature (°C)	DoF		
		9		
		*t*-Stat	*p* Value	Remarks
NF1	25	−2.3	0.04	Significant
NF2		−7.1	5.45 × 10^−5^	Significant
NF3		−5.2	5.29 × 10^−5^	Significant
NF1	30	−7.6	3.02 × 10^−5^	Significant
NF2		−11.1	1.45 × 10^−6^	Significant
NF3		−6.1	1.69 × 10^−4^	Significant
NF1	40	−8.6	1.12 × 10^−5^	Significant
NF2		−5.2	0.0005	Significant
NF3		−2.1	0.06	Not Significant
NF1	50	3.3	0.0082	Significant
NF2		−4.3	0.0018	Significant
NF3		−3.9	3.27 × 10^−3^	Significant

**Table 6:** Percentage (%) enhancement in thermal conductivity as compared to EG.

Samples	Vol %	Temperature (°C)			
		25	30	40	50
		Thermal Conductivity Enhancement [%]			
NF1	0.005	2.3396	4.3512	11.1458	4.4874
NF2	0.0075	4.8003	4.0348	7.4305	4.9903
NF3	0.01	3.6748	3.28322	3.5588	6.5764

## Data Availability

The data presented in this study are available on request from the corresponding author.
